# NRF2 Regulates Cystathionine Gamma-Lyase Expression and Activity in Primary Airway Epithelial Cells Infected with Respiratory Syncytial Virus

**DOI:** 10.3390/antiox11081582

**Published:** 2022-08-16

**Authors:** Mohammad Jamaluddin, Aline Haas de Mello, Nisha Tapryal, Tapas K. Hazra, Roberto P. Garofalo, Antonella Casola

**Affiliations:** 1 Department of Pediatrics, The University of Texas Medical Branch, Galveston, TX 77555, USA; 2 Department of Internal Medicine, The University of Texas Medical Branch, Galveston, TX 77555, USA; 3 Department of Microbiology and Immunology, The University of Texas Medical Branch, Galveston, TX 77555, USA

**Keywords:** CSE, RSV, NRF2, small airway epithelial cells

## Abstract

Cystathionine-y-lyase (CSE) is a critical enzyme for hydrogen sulfide (H_2_S) biosynthesis and plays a key role in respiratory syncytial virus (RSV) pathogenesis. The transcription factor NRF2 is the master regulator of cytoprotective and antioxidant gene expression, and is degraded during RSV infection. While some evidence supports the role of NRF2 in CSE gene transcription, its role in CSE expression in airway epithelial cells is not known. Here, we show that RSV infection decreased CSE expression and activity in primary small airway epithelial (SAE) cells, while treatment with *tert*-butylhydroquinone (tBHQ), an NRF2 inducer, led to an increase of both. Using reporter gene assays, we identified an NRF2 response element required for the NRF2 inducible expression of the CSE promoter. Electrophoretic mobility shift assays demonstrated inducible specific NRF2 binding to the DNA probe corresponding to the putative CSE promoter NRF2 binding sequence. Using chromatin immunoprecipitation assays, we found a 50% reduction in NRF2 binding to the endogenous CSE proximal promoter in SAE cells infected with RSV, and increased binding in cells stimulated with tBHQ. Our results support the hypothesis that NRF2 regulates CSE gene transcription in airway epithelial cells, and that RSV-induced NRF2 degradation likely accounts for the observed reduced CSE expression and activity.

## 1. Introduction

Hydrogen sulfide (H_2_S) is an endogenous gaseous transmitter which participates in the regulation of the physiological functions of the airway, and in the pathophysiology of diseases, such as chronic obstructive pulmonary disease, asthma, pulmonary fibrosis, and hypoxia-induced pulmonary hypertension, as it modulates airway constriction, pulmonary circulation, cell proliferation/apoptosis, fibrosis, oxidative stress, and inflammation (reviewed in [1]). In the past few years, we have shown that H_2_S exerts important antiviral and anti-inflammatory activity in the context of respiratory syncytial virus (RSV) infection [2,3], a primary cause of bronchiolitis and pneumonia in children [4,5]. The cystathionine gamma-lyase (CSE) enzyme is a major producer of H_2_S and CSE-deficient mice show increased RSV replication, and greater disease and inflammatory mediator production compared to CSE-competent mice [2]. We have also shown that RSV infection is associated with decreased levels of H_2_S cellular production in vitro, though CSE-specific enzymatic activity was not measured [3], and with decreased expression of CSE in the lungs of infected mice [2].

CSE is an inducible gene in many types of cells and tissues, and several factors have been shown to regulate its expression. Two consensus specificity protein 1 (Sp1) binding sites have been identified in the core promoter region of the human CSE gene, and Sp1 activation in response to a variety of stimuli induces CSE mRNA expression by binding to the human CSE promoter, as demonstrated by chromatin immunoprecipitation (ChIP) assay (reviewed in [6]). The CSE gene is also modulated by oxidative stress, as hydrogen peroxide induces CSE protein and mRNA expression in human A549 cells through enhanced CSE transcription [6]. Nuclear factor erythroid 2-related factor 2 (NRF2) is an important redox-responsive protein that helps protect the cells from oxidative stress and injury by regulating the expression of antioxidant enzyme (AOE) genes in part through *cis*-acting antioxidant responsive element (ARE) sequences [7]. In vitro studies of mouse embryonic fibroblasts (MEFs) have shown that NRF2 regulates the basal and inducible expression of CSE [8], consistent with the recent observation that rat CSE is induced by platelet-derived growth factor in an NRF2-dependent manner [9]. In the past few years, we have shown that RSV infection induces NRF2 degradation and decreased AOEs expression in cells, mice, and children [10,11,12,13]. The kinetics of reduction in CSE gene expression observed in the lungs of RSV-infected mice [2] mirrors the decrease in NRF2-dependent AOE gene and protein expression, which occurs between day 2 and 3 post infection (p.i.) [12,14], suggesting a causal relationship between NRF2 downregulation and CSE expression in RSV infection. Whether NRF2 regulates human CSE gene transcription in airway epithelial cells and whether it is responsible for decreased CSE expression in the context of RSV is currently unknown. The results of this study identify an NRF2 binding site in the proximal region of the CSE promoter which is necessary for both induction and viral-induced inhibition of the CSE gene in airway epithelial cells.

## 2. Materials and Methods

### 2.1. Cell Culture and Treatment

Human primary small airway epithelial cells (SAE) were purchased from Lonza (Walkersville, MD, USA), and immortalized small airway epithelial (iSAE) cells were established by transducing primary cells with human telomerase and cyclin-dependent kinase-4 retrovirus constructs [15]. Both cells were grown in basal medium supplemented with growth factors, according to the manufacturer’s instructions, in T75-flasks in a humidified incubator with 95% air/5% CO_2_ at 37 °C. At 80–90% confluency, medium was replaced with fresh basal medium without any growth factors and cells were infected with sucrose-purified RSV at a multiplicity of infection (MOI) of three. *tert*-butylhydroquinone (tBHQ) (Sigma-Aldrich, St Louis, MO, USA) was prepared fresh by dissolving in ethanol and diluted in basal medium prior to use.

### 2.2. Preparation of Purified RSV

The human RSV Long strain was grown in Hep-2 cells and prepared by sucrose cushion centrifugation, as described [16]. The viral titer was determined by a methylcellulose plaque assay. Purified RSV aliquots were quick-frozen in dry ice–ethanol and stored at −70 °C until use.

### 2.3. CSE Enzymatic Activity

The assay was performed in 96-well plates as described [17]. Cells were lysed in ice-cold NP40 lysis buffer (1% NP40; 150 mM NaCl; 50 mM Tris-Cl, pH 8.0) for 30 min on ice with the addition of protease inhibitor cocktail. Cell extracts were centrifuged, and supernatants were collected. Twenty microliters of supernatant (50 µg protein) were added to the reaction mixture (50 mM Tris HCl pH 8.0), which was supplemented with 2 mM L-cysteine. Pyridoxal 5′-phosphate (PLP; 10 μM final concentration) was added to the reaction mixture as a cofactor. Finally, an H_2_S-specific fluorescent probe 7-azido-4-methylcoumarin (AzMC, 10 μM) was used to scavenge H_2_S. The mixture was incubated at 37 °C for 2 h, and the fluorescence of the mixture was read at 450 nm emission and 365 nm excitation.

### 2.4. Reverse Transcription-Quantitative PCR (RT-qPCR)

Total RNA was isolated using a RNeasy mini kit (Qiagen, Germantown, MD, USA). On-column DNase digestion was performed using the RNAse-Free DNase Set (Qiagen, Germantown, MD, USA). The synthesis of cDNA was performed with 1 μg of total RNA using iScript Reverse Transcription Supermix (Bio-Rad, Hercules, CA, USA). The cDNA was diluted five times with molecular biology grade water and qPCR amplification was conducted using 2 µL of cDNA, pre-formulated TaqMan Gene Expression Assay (TaqMan MGB probe labeled with FAM and forward and reverse primers, Thermo Fisher Scientific, Waltham, MA, USA), and TaqMan Universal Master Mix (Applied Biosystems, Waltham, MA, USA). The qPCR assays were run in the Bio-Rad CFX Connect Real-Time System. The 18S rRNA was used as a housekeeping gene for normalization. The comparative Ct method (2^−ΔΔCt^) was used to calculate relative quantification.

### 2.5. CSE Luciferase Reporter Vector Construction

Approximately 1 kilobase pair human CSE promoter was cloned from primary SAE cell’s DNA using PCR with a sense primer hybridizing to nucleotides (nt) −973 (5′-AGTC GGTACC GGT GCT AGA AAA CAG CCA CAA TAG-3′ (*Kpn*I site underlined) and an antisense primer hybridizing to nt +119 (5′-AGTC CTCGAG AGA ACA CCG AAG ATA TAA CCA GTC G-3′ (*Xho*I underlined). The PCR fragment was *Kpn*I/*Xho*I-restricted and cloned into the promoter-less firefly luciferase reporter plasmid pGL4 (Promega, Madison, WI, USA). The CSE reporter plasmid was sequenced for authenticity. The 5′-deletion mutations were produced by PCR using −973/+119 CSE-PGL4-luc as the template, CSE +119 nt anti-sense primer and the following sense primers (the *Kpn*I restriction site underlined): for −804, 5′-AGT CGGTACCGCATTTTGTGTAAGGATGTTAGGC-3′; for −355, 5′-AGTCGGTACCCCCGGTTTCAACAC TAAG-3′; for −250, 5′-AGTCGGTACCCTCAGGACGGGTACTC-3′; for −204, 5′-AGT CGGTACCTTGGACCCAGGGACTCCAG-3′; for −162, 5′-AGTCGGTACCCGCGGTGA CGTTTCAG-3′; for −124, 5′-AGTCGGTACCTGTCAGCCAATAAGGAGCG-3′; for −111, 5′-AGTCGGTACCAGGAGCGGGAGACGC-3′. Four blocks of nine base pair site mutations were introduced in the context of the −250/+119 CSE promoter using PCR-directed mutagenesis by overlap extension PCR [18]. Sense mutagenic and antisense mutagenic primers; mutated bases (purine or pyrimidine noncomplementary bases are underlined): for −156 nt mutation (M1), the sense mutagenic primer 5′-CTGGGTGGGGCTGGATCTGTGACGTTTC-3′ and the antisense mutagenic primer 5′-GAAACGTCACAGATCCAGCCCCACCCAG-3′; For −147 nt mutation (M2) the sense mutagenic primer 5′-CGGTCGCGGGTCAGTGTCAGGCAACGC-3′ and the antisense mutagenic primer 5′-GCGTTGCCTGACACTGACCCGCGACCG-3′; for −138 nt mutation (M3), the sense mutagenic primer 5′-TGACGTTTCCGTAGAATCCTCTGCTGTC-3′ and antisense mutagenic primer 5′-GACAGCAGAGGATTCTACGGAAACGTCA-3′; for −129 nt mutation (M4), the sense mutagenic primer 5′-AGGCAACGCCGATTCGTTCAGCCAATA-3′ and antisense mutagenic primer 5′-TATTGGCTGAACGAATCGGCGTTGCCT-3′.

### 2.6. Electrophoretic Mobility Shift Assay (EMSA)

EMSA was performed as described previously with minor modification [19]. Human primary SAE cells were treated with tBHQ (25 µM) for 15 h and nuclear extract was prepared using hypotonic/nonionic detergent lysis. Isolated nuclei were purified by centrifugation through 1.7 M sucrose buffer A for 30 min, at 12,900× *g* and nuclear protein was extracted with buffer C [19]. Proteins were measured by protein assay using bovine serum albumin (BSA) as standard. Nuclear extracts (6–8 µg protein) were incubated with 15 fmol of p32-labeled duplex oligonucleotide probe and 0.3 µg poly dI-dC in a buffer containing 2.5% glycerol, 10 mM Tris-HCl, pH 8.0, 50 mM KCl, 50 mM NaCl, 1 mM MgCl_2_, 1 mM EDTA, 5 mM DTT in a final volume of 10 µL for 15 min at room temperature. HPLC-purified oligonucleotides were used. The sequences of oligonucleotides are shown:

−156/−120 CSE wild type (WT)

5′-CGGTCGCGGTGACGTTTCAGGCAACGCCTCTGCTGT-3′ 

3′-GCCAGCGCCACTGCAAAGTCCGTTGCGGAGACGACA-5′

−156/−120 CSE mutant (M)

5′-CGGTCGCGGGTCAGTGTCAGGCAACGCCTCTGCTGT-3′ 

3′-GCCAGCGCCCAGTCACAGTCCGTTGCGGAGACGACA-5′

Commercially available purified human recombinant NRF2 (rNRF2) (Novus Biologicals, Littleton, CO, USA) was used as a positive control. Complexes were fractionated on 6% native polyacrylamide gels in 0.3× TBE, dried, and exposed to phosphoimager screen overnight.

### 2.7. Cell Transfection

Transfection was carried with X-tremeGnene DNA transfection reagent according to the manufacturer’s instruction. Briefly, iSAE cells were plated in a 24-well plate (50 × 10^3^ cells/well) the day before transfection. Firefly luciferase reporter plasmids (125–250 ng/well) and Renilla luciferase plasmids (pRL-CMV) (50 ng/well) were aliquoted into 1.5 mL microfuge tubes containing 200–400 µL OptiMEM. The contents were mixed and incubated for 5 min at room temperature. X-tremeGENE DNA transfection reagent (Roche/Sigma-Aldrich, St Louis, MO, USA) was added to the reaction mixture at the ratio of 1:3 (1 µg plasmid:3 µL transfection reagent) and incubated for 30 min at room temperature. Each well received equal amounts of plasmids adjusted with empty vector. The mixture was added dropwise to the wells containing fresh basal medium. After 48 h of transfection, cells were harvested, and luciferase activity was measured using dual luciferase assay kit (Promega, Madison, WI, USA).

### 2.8. Chromatin Immunoprecipitation (ChIP)

ChIP assays were performed using a two-step cross-linking protocol, as described in Tian et al. [20]. At the chosen timepoints after infection or treatment, cells were treated with freshly prepared 2 mM disuccinimidyl glutarate (DSG). The subsequent steps were performed using the ChIP-IT High Sensitivity kit (Active Motif, Inc, Carlsbad, CA, USA) following the manufacturer’s instructions with slight modifications. Briefly, after three washes with phosphate-buffered saline (PBS), protein–DNA fixation was conducted with 1% freshly prepared formaldehyde solution for 15 min at room temperature. After cross-linking, cells were harvested and lysed using a Dounce homogenizer to release nuclei. Chromatin was then sonicated to obtain fragments within 200–1200 base pair range. A sample of the chromatin was used to generate the input DNA and the DNA concentration of each pre-IP chromatin preparation was determined. Chromatin (25 µg) was immunoprecipitated with 8 µg of anti-NRF2 rabbit polyclonal antibody (MBL International, Woburn, MA, USA). A sample immunoprecipitated with 8 µg of Normal Rabbit IgG (Cell Signaling Technology, Danvers, MA, USA) was used as negative control. After IP, cross-links were reversed, proteins were digested with Proteinase K, and DNA was purified. The enrichment of ChIP DNA was analyzed by qPCR using Universal SYBR Green Fast qPCR (ABclonal Technology, Woburn, MA, USA) and ChIP primers spanning the CSE gene proximal promoter region (−150 to −5 from the TSS). The sequence of ChIP primers was 5′-CAGGGACTAACACCACTTGGACCCAG-3′ (sense), 5′-TTATTAGCGGGTCTGCAGTCTCACGATC-3′ (anti-sense). The ChIP primer sequence of NAD(P)H quinone dehydrogenase 1 (NQO1) promoter was 5′-GTGGAAGTCGTCCCAAGAGA-3′ (sense), 5′TGTCTCCCCAGGACTCTCTCAG-3′ (anti-sense). The qPCR assays were run in the CFX Connect Real-Time System (Bio-Rad, Hercules, CA, USA). Results were expressed in fold change relative to IgG [20].

### 2.9. Statistical Analysis

The data were analyzed by GraphPad Prism software version 9 (San Diego, CA, USA). All results are expressed as mean ± SEM. Results were compared between two groups by Student’s *t*-test. Results were compared among treatment groups by either one-way or two-way ANOVA followed by Tukey’s test. Results were considered significant at *p* < 0.05.

## 3. Results

### 3.1. CSE Enzymatic Activity and Expression in Response to RSV Infection and tBHQ Treatment

**Figure 1:**
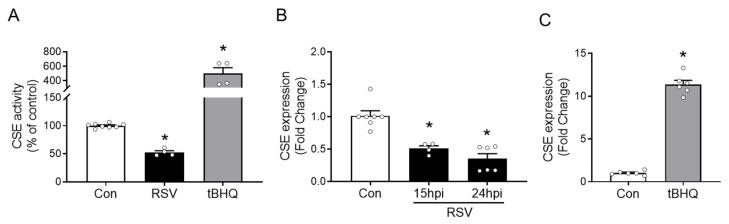
CSE activity and expression in response to respiratory syncytial virus (RSV) infection and *tert*-butylhydroquinone (tBHQ) treatment. SAE cells, either control (Con), infected with RSV for 24 h, or treated with tBHQ (25 µM) for 15 h, were harvested to measure CSE enzymatic activity, and expressed as percentage of control, as described in Materials and Methods (**A**). CSE gene expression, expressed as fold change over control, was measured in RSV-infected SAE cells at 15 and 24 h post infection (p.i.) (**B**) or in response to tBHQ (25 µM) for 15 h by RT-qPCR (**C**). Results are expressed as mean ± SEM of combined data from two independent experiments (each circle represents individual data). * *p* < 0.05 relative to control cells.

We have previously shown that the RSV infection of A549 cells, a carcinoma-derived cell line retaining the features of type II alveolar cells, was associated with decreased H_2_S production [3], although we did not determine whether this was specifically due to the inhibition of CSE, the major H_2_S-producing enzyme in epithelial cells. To determine whether this was the case, SAE cells were either infected with RSV or treated with tBHQ, an antioxidant able to induce NRF2 activation [12,21] as positive control. Cells were harvested at different time points to measure CSE enzymatic activity and expression. RSV infection resulted in a 50% reduction of CSE enzymatic activity compared to the control (uninfected) cells, while tBHQ treatment was associated with a 400% increase over the control (Figure 1A). Next, we quantified the CSE mRNA level by RT-qPCR in RSV-infected or tBHQ-treated SAE cells. RSV significantly inhibited CSE gene expression in a time-dependent manner (Figure 1B), while tBHQ led to a 10–12-fold increase of CSE mRNA levels in treated SAE cells compared to the control (Figure 1C).

### 3.2. Effect of RSV Infection and tBHQ Treatment on CSE Promoter Activity

**Figure 2:**
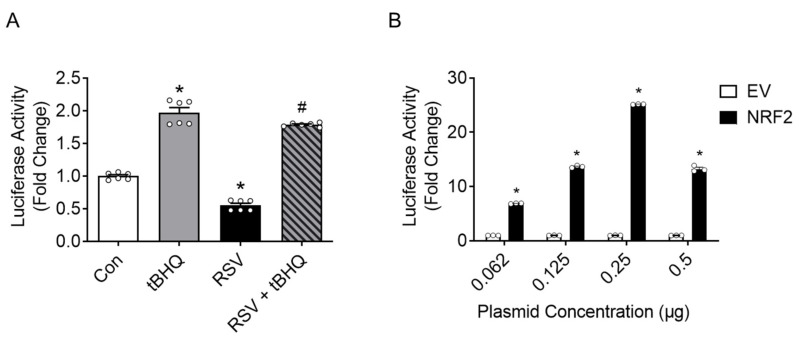
CSE promoter activity in response to RSV infection, tBHQ stimulation and NRF2 expression. iSAE cells were transfected with -973 CSE-PGL4-Luc and Renilla-Luc plasmid (pRL-CMV) in 24-well plates. After 24 h, transfected cells were left untreated (control) or treated with tBHQ (25 µM) for 15 h or infected with RSV for 24 h alone or in combination with tBHQ (RSV + tBHQ). Luciferase activity was normalized to Renilla luciferase activity. Results are expressed as mean ± SEM of triplicate determination of a representative experiment repeated twice and expressed as fold change relative to control. * *p* < 0.05 relative to control cells, # *p* < 0.05 compared to RSV infected cells (**A**). iSAE cells were co-transfected with increasing amounts of NRF2 expression vector or the corresponding empty vector and harvested 48 h post transfection to measure luciferase activity. Luciferase activity was normalized to Renilla luciferase activity. Results are expressed as mean ± SEM of triplicate determination of a representative experiment repeated twice and expressed as fold change relative to control. * *p* < 0.05 relative to empty vector (**B**). Each circle represents individual data.

To investigate the possible contribution of NRF2 in the transcriptional regulation of the CSE gene in human airway epithelial cells, we used reporter gene assays. Approximately 1 kilobase of the proximal human CSE promoter was cloned into the PGL4.1-Luc vector to create our full-length CSE-PGL4_luc reporter expression vector (−973 CSE-PGL4_luc). We initially examined the effect of RSV infection and tBHQ treatment on CSE promoter-driven luciferase activity in iSAEs. Cells were transfected with −973 CSE-PGL4_luc reporter plasmid and 24 h later they were either treated with vehicle (control) or tBHQ for 15 h, or infected with RSV for 24 h. tBHQ treatment increased luciferase activity about 2-fold, while RSV infection decreased basal luciferase activity to half, when compared to the control cells (Figure 2A). The addition of tBHQ 1 h after RSV infection (RSV + tBHQ) was able to rescue the RSV-induced inhibition of CSE-driven luciferase activity, similar to what we have previously shown for antioxidant gene expression and ARE-dependent gene transcription following RSV infection [12]. As these results supported the hypothesis that NRF2 might be involved in the reversal of the transcriptional regulation of the CSE gene, we next examined the CSE reporter activity in response to increasing concentrations of pCI-HA-NRF2 expression vector or corresponding empty vector. NRF2 expression vector (0.062 µg to 0.25 µg/well) was associated with a dose-dependent increase in luciferase activity from 5-fold to 25-fold over the empty vector transfected to cells with the same concentrations (Figure 2B).

### 3.3. Effects of 5’ Deletions and Site Mutations of the CSE Promoter Sequence on NRF2-Inducible Activity

**Figure 3:**
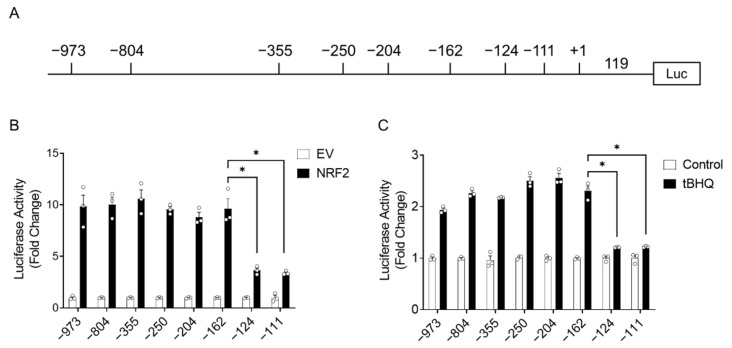
Effects of 5’ deletions in the CSE promoter sequence on NRF2-inducible activity. Schematic representation of the CSE promoter deletion constructs. Numbering is relative to transcription initiation site (+1) (**A**). iSAE cells were co-transfected with the CSE-PGL4-Luc deletion mutants and Renilla-Luc plasmid plus either the empty vector or the NRF2 expression vector and harvested 48 h later to measure luciferase activity. Luciferase activity was normalized to Renilla luciferase activity. Results are expressed as mean ± SEM of triplicate determination of a representative experiment repeated twice and expressed as fold change relative to empty vector. * *p* < 0.05 relative to −973-PGL4-Luc (**B**). iSAE cells were co-transfected with the CSE-PGL4-Luc deletion mutants and Renilla-Luc plasmid for 24 h. Cells were left untreated or treated with tBHQ (25 µM) for 15 h before harvesting to measure luciferase activity. Luciferase activity was normalized to Renilla luciferase activity. Results are expressed as mean ± SEM of triplicate determination of a representative experiment repeated twice and expressed as fold change relative to control. * *p* < 0.05 relative to control cells (**C**). Each circle represents individual data.

To locate the potential NRF2 binding site(s) on the proximal CSE promoter, a promoter deletion strategy was undertaken. Eight deletion mutations of −973 CSE-PGL4_luc plasmid were generated, as shown in Figure 3A. Cells were co-transfected with each mutant plasmid and the NRF2 expression vector or empty vector. We observed that NRF2-stimulated luciferase activity was significantly reduced in cells transfected with −124 CSE-PGL4_luc and −111 CSE-PGL4_luc indicating that an NRF2 binding site may be located between −162 and −124 (nt) of the CSE promoter (Figure 3B). tBHQ treatment also failed to stimulate luciferase activity in cells transfected with −124 CSE and −111 CSE-Luc plasmids (Figure 3C), supporting the hypothesis that an NRF2 response element could be located between nt −162 and −124 of the CSE promoter.

**Figure 4:**
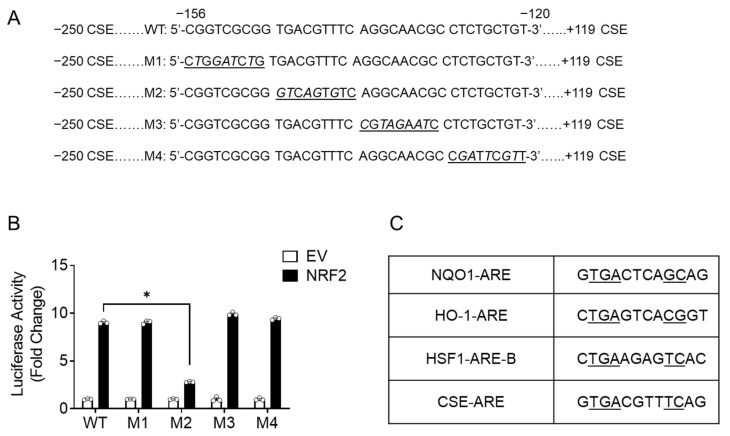
Effects of site mutations in the CSE promoter sequence on NRF2-inducible activity. Schematic representation of the CSE promoter site mutation constructs. Numbering is relative to transcription initiation site. Four segments of nine nucleotides for each of the 36 base pair regions of the CSE promoter comprised between nt −156 and −120 were mutated separately (mutation sites shown in italic underlined) (**A**). iSAE cells were co-transfected with the wild type (WT) −250-CSE-PGL4-Luc or the site mutants (M1 to M4) and Renilla-Luc plasmid plus either the empty vector or the NRF2 expression vector and harvested 48 h later to measure luciferase activity. Luciferase activity was normalized to Renilla luciferase activity. Results are expressed as mean ± SEM of triplicate determination of a representative experiment repeated twice and expressed as fold change relative to empty vector (each circle represents individual data). * *p* < 0.05 relative to WT PGL4-Luc (**B**). Nucleotide comparison of putative CSE antioxidant response element (ARE) with canonical (NQO1) and non-canonical (heme oxigenase-1, HO-1 and HSF1) ARE sites (**C**).

To narrow it down to a specific site, we introduced purine to pyrimidine mutations in four segments of nine nucleotides for each of the 36 base pair regions of the CSE promoter comprised between nt −156 and −120, using a −250CSE/+119-Luc template. We did not modify the nucleotides between −162 and −156 of the promoter, as that region contains a Sp1 binding site [22] (Figure 4A). Cells were co-transfected with the wild type (WT) or the mutated plasmids (defined as M1 to M4) and the NRF2 expression vector or the empty vector. We observed that luciferase activity was significantly reduced with the M2 plasmid (Figure 4B), to the same extent of promoter deletion to −124 nt (Figure 3B). Sequence alignment with the consensus NRF2 binding site, defined as ARE, of the well-known NRF2 gene target NQO1 identified a 50% sequence similarity at the 5′ end (GTGAC), and an even higher similarity with non-canonical ARE sites of NRF2-regulated genes such as heat shock factor 1 (HSF1) (Figure 4C).

### 3.4. NRF2 Binding to CSE Promoter by EMSA and ChIP Assay

**Figure 5:**
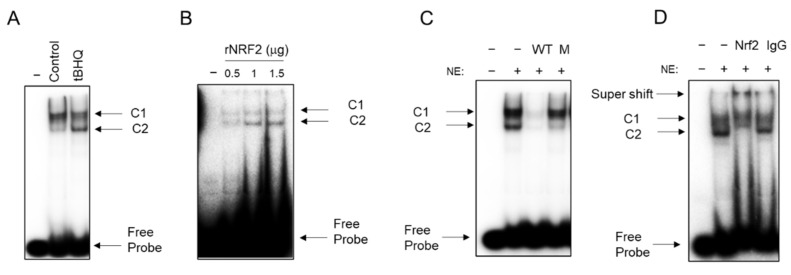
EMSA of CSE putative ARE binding complexes in response to tBHQ treatment. Nuclear extracts (6 µg) from control and SAE cells treated with tBHQ (25 µM) for 15 h were used in EMSA. Two DNA–protein complexes, C1 and C2, are detected in control cells. C2 binding is further increased by tBHQ treatment (**A**). Similar complex formation is observed with purified recombinant NRF2 (rNRF2) (**B**). Competition assay using wild type (WT) and mutant (M) oligos (50×) and nuclear extracts of tBHQ-treated SAE cells (**C**). Super-shift assay with specific anti-NRF2 antibody and control IgG using nuclear extracts of tBHQ-treated SAE cells (**D**).

To investigate whether NRF2 could bind to the −156 to −120 CSE promoter region, EMSA was carried out with nuclear extract from control and tBHQ-treated SAE cells. The EMSA results showed that there were two bound complexes, C1 and C2, with C2 being inducible by tBHQ treatment (Figure 5A). Similar C1 and C2 complexes were formed with purified recombinant NRF2 (rNRF2) (Figure 5B). To confirm the specificity of the C1 and C2 complexes, we performed competition assays using tBHQ-treated SAE cell nuclear extracts and with wild type and mutant oligos (M), the latter containing the same mutations present in the M2 CSE-PGL4_luc plasmid that greatly reduced NRF2 inducibility of luciferase activity. Both complexes were sequence specific, as demonstrated by their competition with unlabeled wild type but not mutant oligonucleotide (Figure 5C). To confirm the presence of NRF2 in the DNA–protein complexes, we performed super-shift assays with anti-NRF2 antibody or IgG control. Both C1 and C2 complexes were shifted by the specific antibody but not IgG (Figure 5D), confirming that NRF2 specifically bind to the −156/−120 CSE promoter region containing the identified ARE-like binding site.

**Figure 6:**
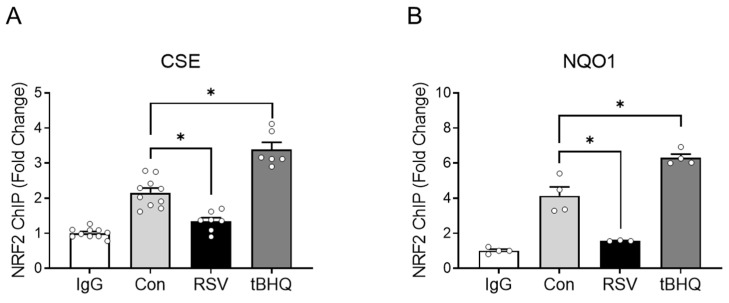
ChIP assay of endogenous CSE and NQO1 promoters. Chromatin from primary SAE cells infected with RSV for 20 h or treated with 25 µM tBHQ for 15 h was immunoprecipitated with anti-NRF2 antibody or IgG as negative control. qPCR was performed with immunoprecipitated DNA using primers spanning either the putative ARE site of the CSE promoter (**A**) or the NQO1 promoter (**B**). Fold change was calculated compared to IgG control. Data are expressed as mean ± SEM of three or four replicate data from two independent experiments (each circle represents individual data). * *p* < 0.05 relative to control cells.

Finally, to confirm the binding of NRF2 to the endogenous CSE promoter, ChIP assays were conducted with SAE cells either untreated, infected with RSV, or treated with tBHQ. Cells were subjected to a two-step cross-linking, as described above, and an equal amount of chromatin was immunoprecipitated with anti-NRF2 antibody or IgG control. The qPCR analysis of bound DNA with specific primers recognizing the CSE promoter region spanning −150 to −5 nucleotides demonstrated that RSV infection induced a 50% reduction in NRF2 binding to the CSE promoter, compared to binding in control cells, while tBHQ treatment was associated with a little over 1.5-fold increase (Figure 6A). DNA from the same experiment was subjected to qPCR with validated primers specific to human NQO1 promoter, as a positive control for this experiment. Our results demonstrated that there was a higher basal binding of NRF2 to the NQO1 promoter in comparison to the CSE one, with a similar decrease in NRF2 binding following RSV infection and an increase upon tBHQ treatment observed for NRF2 binding to the CSE promoter (Figure 6B). These results parallel the ones found for CSE mRNA expression (Figure 1B,C) and reported gene assays (Figure 2A) following both stimuli, supporting the hypothesis that NRF2 regulates CSE expression in airway epithelial cells and that RSV-induced NRF2 degradation is responsible for the inhibition of CSE gene transcription, similar to what we have observed for AOE expression in epithelial cells [12].

## 4. Discussion

CSE and its product, H_2_S, play important roles in the physiological and pathophysiological functions of the respiratory system. Our past studies have demonstrated for the first time the antiviral and anti-inflammatory role of CSE/H_2_S both in vitro and in vivo, following in RSV infection [2,3]. The expression of CSE has not been extensively investigated, but it is known to be regulated by oxidative stress, as in the case of H_2_O_2_ cell stimulation [23]. The transcription of many oxidative stress-inducible genes is regulated in part through *cis*-acting ARE sequences. This element has been identified in the regulatory regions of genes encoding detoxification enzymes, such as NQO1, as well as many AOE. The transcription factor NRF2 is well known for its ability to reduce oxidative stress by activating numerous antioxidant genes. In response to oxidative stress, NRF2 is activated and binds to gene promoter ARE sequences inducing the ARE-mediated gene transcription of many antioxidant genes [24,25]. An initial study from Hourihan, Kenna and Hayes [8] demonstrated that treatment with H_2_S pharmacologic donors failed to induce CSE expression in NRF2-deficient mouse fibroblasts. Our own study in NRF2-deficient mice found that the level of CSE expression was decreased compared to wild type mice in response to RSV infection [26]. These findings strongly suggest that NRF2 is a potential transcriptional regulator of CSE expression. However, whether NRF2 regulates human CSE gene transcription in airway epithelial cells and whether it is responsible for changes in CSE expression in the context of RSV was not known. In the present study, using primary SAE cells, we confirmed that RSV infection is associated with decreased CSE expression and enzymatic activity (Figure 1). Using reporter gene assays, we demonstrated that luciferase activity driven by a CSE promoter was inhibited by RSV infection but was activated by the antioxidant tBHQ (Figure 2). tBHQ causes the stabilization and activation of NRF2 by promoting the dissociation of NRF2 from its cytoplasmic inhibitor Keap1, leading to NRF2 nuclear translocation and binding to the ARE sequence of antioxidant genes [27]. The RSV-mediated inhibition of CSE-driven luciferase activity was reversed by tBHQ treatment, similar to what we have previously shown for ARE-dependent gene transcription and AOE expression, both in vitro and in vivo, due to the restoration of NRF2 nuclear levels [12] (Figure 2).

An initial analysis of the proximal human CSE promoter using a TFBIND program [28] did not locate a canonical ARE site; therefore, we made a series of deletions and site mutations of the CSE proximal promoter and identified a region between −162 and −124 nt that conferred inducibility upon NRF2 stimulation, with the ARE site located between −147 and −138 nt of the CSE promoter (Figure 3 and Figure 4). After the alignment of the CSE promoter putative ARE-containing sequence with other known ARE sequences, we found that there was a significant sequence homology with both canonical ARE sites, such as the one of the NQO1 promoter, and non-canonical sites such as the one of the HSF1 promoter. In vitro binding assays (EMSA) and ChIP assays of the endogenous CSE promoter identified regulated NRF2 binding to the region containing the putative ARE site (Figure 5 and Figure 6). The EMSA result showed two bound complexes, C1 and C2, with C2 being inducible by tBHQ treatment, using a probe containing the putative ARE-like site, with similar complexes formed with purified recombinant NRF2 (Figure 5). NRF2 heterodimers with the small MAF (musculoaponeurotic fibrosarcoma) family of transcription factors, and NRF2 alone does not bind to ARE as strongly [29], which would explain the lower levels of C1 and C2 complexes formed on the probe in spite of the high amount of NRF2 used for the assay.

Our study is the first to identify an ARE-like site on the CSE proximal promoter that regulates basal and inducible gene levels through NRF2. Although the ARE core sequence 5′-puGTGACNNNGC-3′ (N represents any nucleotide) has been considered the canonical NRF2 binding site for many genes involved in detoxification and antioxidant responses [24,25], several studies have identified other ARE-like sequences, to which NRF2 binds and regulates the expression of several genes. For example, Nioi et al. [30] have identified a novel NRF2 ARE in the mouse NQO1 gene which is different from consensus ARE and binds NRF2 at higher levels in response to sulforaphane. Under oxidative stress conditions, NRF2 has been shown to activate HSF1 through a non-canonical ARE site as well [31]. Our results convincingly show NRF2 binding to the endogenous CSE promoter that parallels levels of its activation in SAE cells, with decreased binding in response to RSV infection, which leads to NRF2 degradation, and increased binding following tBHQ treatment, which activates NRF2 (Figure 6). Similar changes in NRF2 binding to the endogenous NQO1 promoter were observed after both treatments.

A previous study has identified Sp1 as a critical regulator of CSE expression in human smooth muscle cells [22]. It identified three consensus Sp1 binding sites in the CSE core promoter spanning nt-226 and -41, two of which were necessary for basal CSE promoter activation. The mutation of the Sp1 site located at −161 of the CSE promoter did not affect NRF2-stimulated luciferase activity compared to wild type (data not shown). As CSE promoter inducibility by NRF2 was lost with the −124 nt deletion, the second Sp1 site located at -61, which is required for basal CSE promoter activation [22], could not have contributed to NRF2 inducibility as well.

In summary, we provide evidence that NRF2 has a major role in the regulation of CSE in primary airway epithelial cells. NRF2 directly binds to the CSE promoter and regulates its basal and inducible activity. As H_2_S production by CSE is critical for controlling RSV replication and inflammatory responses, maintaining NRF2 cellular levels during the course of RSV infection could prove beneficial in modulating the severity of lung disease.

## Data Availability

Data is contained within the article.
